# Free Radical-Scavenging Capacities, Phenolics and Capsaicinoids in Wild Piquin Chili (*Capsicum annuum* var. *Glabriusculum*)

**DOI:** 10.3390/molecules23102655

**Published:** 2018-10-16

**Authors:** Yolanda del Rocio Moreno-Ramírez, Guillermo C. G. Martínez-Ávila, Víctor Arturo González-Hernández, Cecilia Castro-López, Jorge Ariel Torres-Castillo

**Affiliations:** 1 Institute of Applied Ecology, Autonomous University of Tamaulipas, Gulf Division 356, Ciudad Victoria, 87019 Tamaulipas, Mexico; ydelrocio_moreno@hotmail.com; 2 Laboratory of Chemistry and Biochemistry, School of Agronomy, Autonomous University of Nuevo Leon, General Escobedo, 66050 Nuevo Leon, Mexico; guillermo.martinezavl@uanl.edu.mx (G.C.G.M.-A.); caslopcec28@hotmail.com (C.C.-L.); 3 Posgrado de Recursos Genéticos y Productividad-Fisiología Vegetal, Colegio de Postgraduados, Texcoco, 56230 Estado de Mexico, Mexico; vagh@colpos.mx

**Keywords:** chili, capsaicinoids, phenolics, free radical-scavenging, geographical variation

## Abstract

The total phenolic compounds content, free radical-scavenging capacity and capsaicinoid content in populations of wild Piquin chili (*C. annuum*) were studied. Aqueous and hydroalcoholic extracts from nine ecotypes were evaluated. High contents of phenolic compounds and free radical-scavenging capacities were observed for both extracts; however, the values that were found for the hydroalcoholic phase were substantially higher. LC-MS analysis allowed for the detection of 32 compounds, where apigenin-8-*C*-glucoside followed by vanillic acid 1-*O*-β-*o*-glucopyranosylester (Isomer I or II) and 7-ethoxy-4-methylcoumarin were the most widely distributed; they were found in more than 89% of the ecotypes. The diversity of identified phenolic compounds was different among ecotypes, allowing them to be distinguished by chemical diversity, free radical-scavenging capacities and heat Scoville units. The total capsaicinoid content was higher in Population I (23.5 mg/g DW) than in Populations II and III, which had contents of 15.3 and 10.7 mg/g DW, respectively. This variability could lead to phytochemical exploitation and the conservation of the natural populations of wild chili.

## 1. Introduction

The natural variation of phytochemicals is an ecologically and evolutionarily important characteristic for plant plasticity responses when they are faced with different environmental challenges [1]. These are natural compounds that give plants their basic organoleptic characteristics, such as color, flavor, and aroma and they are also associated with antioxidant, free radical-scavenging, prebiotic, and medicinal effects, especially the prevention of diseases, such as diabetes and hypertension [2]. Therefore, these substances could be responsible for the beneficial effects associated with plants and have a direct impact on quality and consumer preferences [3]. 

A large number of studies have examined bioactive compounds and their diversity within the genus *Capsicum*; most studies have focused on domesticated species [4]. Variability in the expression and accumulation of metabolites (phenolic compounds, carotenoids, and capsaicinoids) is associated with adaptations to the local environment where the genotype developed [5]. The morphological, genetic, and phytochemical diversity of *C. annuum* [6] has been dependent on human selection, management, and exploitation of its characteristics. For this reason, morphotypes satisfy the requirements of different applications such as type, color, shape, ripeness, and pungency, which depend on their phytochemical profiles [7] and determine their broad culinary, pharmacological, and industrial utility. 

Similar patterns of use have been observed in wild chili and primarily in chilis with culinary and therapeutic applications. Nevertheless, few studies have analyzed the composition of metabolites in *C. annuum* var. *glabriusculum* [8,9,10], which are mainly focused on the variation and quantitation of capsaicinoids, because they are responsible for pungency and are also associated with chemopreventive roles [11]). However, there is a substantial lack of knowledge regarding their biological and eco-nutritional potential despite the great variability observed in their flavors and therefore in their compositions and phytochemical concentrations. Fruits of Piquin chili, as a wild resource, present wide variation in metabolite contents that are produced by environmental, genetic, phenological, and processing conditions, which encourages the determination of the responsible phytochemicals for biofunctional and nutritional characteristics. Variations in acidity, color, and pungency have been reported during the ripening process, similar to that observed during processing for consumption (drying and pickling). Additionally, the contents of antioxidants and capsaicinoids showed variations in natural conditions but also during processing [8,9,10,12,13]. This highlights the need to know the identity, quantity, and diversity of phytochemicals in the fruits to select better conditions for preserving the nutritional potential and organoleptic characteristics for direct consumption or for industrial purposes.

Previously, variation in the capsaicinoid content of Piquin chili was studied and related to climatic conditions and vegetation; however, despite detailed descriptions of climate conditions, other compounds were not identified or quantified [13]. Because this is a wild plant genetic resource, it is important to describe traits for breeding programs and to develop efficient management and use strategies. For this reason, exploration of the bioactive phytochemical constituents of wild chilis may help to elucidate the compositional expression in different environments (micro niches) where the plants develop and are harvested. To understand how the natural growing conditions could affect phytochemical accumulations on this species, we studied the phenolic content, free radical-scavenging capacity, and capsaicinoid content of Piquin chili (*C. annuum* var. *glabriusculum*) from different geographic areas.

## 2. Results and Discussion

### 2.1. Total Phenolic Content and Free Radical-Scavenging Capacity

The samples that were analyzed in this study were from wild native populations that grew in distinct geographic situations. Fruit gathering was performed in the same manner as the conventional practices used by local gatherers. Therefore, the methods largely represent the common handling conditions prior to consumption. Previously, the capsaicinoid contents of some samples of Piquin chili were reported. However, the ripening stage of fruits and processing were not specified [13]; this is relevant information due to its impact on the quantitation of metabolites.

Variations dependent on the extraction solvent, geographic origin of the Piquin population and the ecotype (within populations) were observed in TPC (total phenolics content) and levels of free radical-scavenging capacity. Statistical analysis showed highly significant differences (*p* < 0.01) among extraction solvents for all free radical-scavenging parameters evaluated; in particular, the extraction with ethanol showed the highest values of TPC (381 ± 113.1 mg GAE/g DW (Gallic acid equivalents/grams of dry weight)), DPPH^•^ (2,2-diphenyl-1-picrylhydrazyl radical) (70.3 ± 19.6 mM TE/g DW (Trolox equivalents/grams of dry weight), and ABTS^•+^ (2,2′-azino-bis (3-ethylbenzothiazoline-6-sulphonic acid radical) (180.5 ± 74.0 mM TE/g DW) with respect to those obtained when using water as solvent, whose values were 185.8 ± 76.9 mg GAE/g DW in the case of TPC, 13.8 ± 9.6 mM TE/g DW for DPPH^•^ and 89.3 ± 52.0 for ABTS^•+^ mM TE/g DW. 

Population II had the highest TPC and ABTS^•+^ inhibition in both extracts and the highest DPPH^•^ inhibition in the hydroalcoholic extraction. The aqueous extracts had the lowest levels of DPPH^•^ inhibition in all the populations, while the samples from Population I and Population III showed similar free radical-scavenging capacities and TPC in both solvents.

When the TPC, DPPH^•^, and ABTS^•+^ inhibition in each ecotype of their respective populations were analyzed, statistically significant differences (*p* ≤ 0.05) were observed, and when the obtained intervals were compared, a very broad, obvious intra-population differentiation could be seen (Table 1). Although the samples within the ecotypes of each population were generally similar, some samples showed noticeably high values in both solvents.

The ecotypes from Population III were different from those of the other populations, III-1 had the highest TPC and inhibition of the ABTS^•+^ radical; however, differences between the two ecotypes were observed in DPPH^•^ radical inhibition depending on the extraction solvent. This finding suggests that the variations in the TPC levels and DPPH^•^ and ABTS^•+^ inhibition were dependent on the solvent and are associated with geographic origin, but, according to the intra-population variation observed, they are independent of the ecotype within each population. 

The TPC levels detected in this study indicate broad variations among ripe fruits within wild populations. Thus, it is important to consider that local environmental differences and genetic variation influence the accumulation of TPC and free radical-scavenging, as has been reported for other metabolites and other species [14] The variations among different morphotypes of *C. annuum* from different locations have been reported with ranges from 69.7 to 350.4 mg EAG/100 g fresh fruit depending on the morphotype [15]. Moreover, the TPCs found in the samples used in this study were similar to the contents that were found in *C. annuum*, L. var. *Hungarian* fruits subjected to different types of drying even when the temperatures used were different. For this reason, we emphasize that postharvest handling impacts the contents of these bioactive compounds [16].

The free radical-scavenging capacities of the aqueous and hydroalcoholic extracts were determined and were consistent with previous reports [17]. Differences in phytochemical detections could be mainly attributed to polarity variations between water and the alcoholic solution. Phytochemical patterns from plant samples were influenced by the type of solvent, which, according to literature, has a substantial impact on the levels of free radical-scavenging capacities detected. Additionally, the diversity of compounds solubilized is dependent on the polarity of the solvent [18,19,20,21].

The extraction phase has a strong influence on the yields of phenolic compounds as pointed out by Putnik, Bursać, Jezek, Sustić, Zorić and Dragović-Uzelac [22]. The aforementioned studies evaluated methanol and ethanol in different concentrations as solvents for anthocyanins extraction from grape skin, reporting that 70% concentration showed the highest extraction efficiency. Aqueous solvent preparations that were used for phenolics and antioxidants extractions have been shown to be useful when applied to a wide range of plants materials, being superior even with the use of concentrated solvents [23]. Additionally, the use of water as a phase of extraction phase has shown good recovery of phenolics from several materials, for example, those from purple sweet potatoes [24], which supports the potential of both kinds of phases to extract phenolics as done in this research.

High free radical-scavenging capacities were detected in the samples of our study, and they were higher than those of previous reports which indicated total free radical-scavenging capacities between 26.6 and 44.4 μmol TE/g dry tissue [21] in four varieties of *C. annuum*. 

In this study, the levels of phenolic compounds corresponded to the levels of free radical-scavenging capacity for both DPPH^•^ and ABTS^•+^ radicals; that is, the higher the phenolic compounds content, the greater the free radical-scavenging capacity [21]. However, in other cases, the free radical-scavenging capacities and phenolic compounds content are not always correlated [16,17,19,20,21] because of a possible relationship between the phenolic compounds and important chemical interactions, such as antagonism, which can occur due to the presence of several phenolic compounds [25]. For this reason, it is important to consider sample processing and handling as well as extraction and quantification techniques and the nature of the plant material and the prevailing conditions where it grows. 

Moreover, as noted by Durak, Kowalska, and Gawlik-Dziki [26], the TPC identified in the evaluated populations could be used to increase the nutritional value of foods. The concentrations of phenolic compounds and free radical-scavenging capacities identified in the ecotypes of wild Piquin chili are greater than the values determined in apple, quince, chokeberry, cranberry, blackcurrant, and bilberry [27], which are considered free radical-scavenging compounds-containing foods and whose consumption positively and profoundly affects performance and health mainly through a lower incidence of chronic pathologies.

### 2.2. Identification of Compounds by UPLC-ESI-Q/TOF-MSe

When the phenolic compounds of all the samples were analyzed by LC-MS, 32 compounds were detected. Of those, 29 were identified and three were reported as unknown compounds and only their retention times were established (Table 2). The distributions of the compounds varied; some were found in most of the populations and some had limited distribution in at least one ecotype of a population. The most common compound for all of the samples was apigenin-8-*C*-glucoside, followed by vanillic acid 1-*O*-β-*o*-glucopyranosylester (Isomer I or II) and 7-ethoxy-4-methylcoumarin, which were observed in more than 89% of the ecotypes.

All the populations shared nine compounds; Population I and Population II shared three compounds; Population II and Population III shared two compounds; and, Population I and Population III had two compounds in common. The remaining 15 compounds were present in at least one ecotype of each population and were therefore considered compounds of exclusive distribution associated with the geographic origin of the population. 

Eleven compounds were distributed exclusively in Population I, namely genistein-4,7′-dimethylether, ascorbic acid, 1-*O*-galloyl-β-d-glucose, citric acid, caftaric acid, 6′′-*O*-acetyl daidzin, quercetin 3,7-diglucuronide and caffeoyltartaric acid, gallic acid, apigenin-6-*C*-hexoside-8-*C*-pentoside, and benzopyrano [4,3-b] quinoline-6-ones. In the case of Population II, two unknown compounds had exclusive distribution, while in Population III, two compounds of restricted distribution were found, namely citric acid (Isomer II) and spinochrome A. The ecotype I-2 had the highest number of detected compounds, followed by I-1 and II-1; ecotype II-2 was less diverse. In ecotypes I-2 and I-3, the largest number of exclusive distribution compounds was identified; ecotypes I-1, I-4, II-1, II-2, III-1, and III-2 had only one exclusive compound. In contrast, ecotypes II-2 and II-3 did not have any exclusive compounds. 

Overall, by population, the highest number of compounds detected was in Population I, with 80.6% of the detected compounds, followed by Population II and Population III, which contained 48.4% of the compounds, respectively. Population I had a notably different composition due to the number of exclusive distribution compounds, while the other populations had fewer exclusive compounds and more compounds in common with ecotypes of different populations. Regarding unknown compounds, the unknown compound present at the retention time of 5.514 min in both Populations I and II had a [M − H]^−^ of 374.0412, and the unknown compounds present at a retention time of 2.909 min and 4.872 min had a [M − H]^−^ of 233.1501 and 457.1479, respectively; and, they were registered only in ecotypes from Population II. No unknown compounds were detected in the ecotypes of Population III.

We confirmed that there is great diversity among the phenolic compounds in wild Piquin chilis, and some of these compounds have been previously reported. For example, the presence of organic acids, nucleosides, amino acids, derivatives of fatty acids, derivatives of amino acids, derivatives of isoflavones, phenolic acids, coumaroyl, feruloyl and benzyl glucosides as well as quercetin, apigenin, luteolin and kaempferol conjugates in the form of glucosides has been reported in the chemical compositions of *C. annuum* [5]. 

This composition is similar to that of wild Piquin chili, where we found phenolic acids, glucosides, nucleosides, aminoacids, and phenolic derivatives using UPLC-ESI-Q/TOFMS listed in Table 2. Nevertheless, ours is the first report of polygalaxanthone and of spinochrome A. In the first case, polygalaxanthone III was isolated from the roots of *Polygala tenuifolia* and is associated with the diverse bioactivities of plants of the genus *Polygala* [28], and it belongs to a group of xanthonoids that are known for their antioxidant activity. Spinochrome A was reported as a natural pigment isolated from the spines of sea urchin and is a derivate of naphthoquinone [29]; this is the first time that this compound has been reported in chili fruits. 

Some of the identified compounds in the chili samples were associated with antioxidant and free radical-scavenging capacity according to previous reports, some of them with differential contributions to the total free radical-scavenging capacity. Such differential contribution could be related to the chemical structure, number of free hydroxyl groups, solubility, and concentration in the sample. For example, in the identification and quantitative determination of antioxidants from prune, it was observed that some structural-related phenolics presented similar activities. From these, the 4-*O*-caffeoylquinic acid had slightly higher activity against superoxide radicals than that of ascorbic acid [30]; this compound was detected in four ecotypes of Piquin chili. 

On the other hand, the free aromatic amino acids are frequently associated with antioxidant activity. These were detected in eight ecotypes, where tyrosine was detected in seven of those ecotypes. Nevertheless, despite tyrosine being considered as an antioxidant, its capacity against the DPPH^•^ and ABTS^•+^ radicals is lower than those shown by l-DOPA and other antioxidants, including the Trolox [31]. Gallic acid and quercetin were also detected in some Piquin chili samples. Both phenolics are considered as good antioxidants and are used as standards for antioxidant determinations, and although they have different behavior against DPPH^•^ and ABTS^•+^ radicals, they have higher capacities when compared with other standards [32]. 

In contrast to the above-mentioned, the apigenin-8-*C*-glucoside, a phenolic present in all chili ecotypes, has been considered as an agent with no contribution to total antioxidant activity against ABTS^•+^ [33]. This allows us to see that the total free radical-scavenging capacity is a result of the differential contribution of each compound, and that this is a common fact in natural antioxidant activities; in this case, from Piquin chili fruits. The broad heterogeneity of the components, present among the population samples, is likely the result of the influence of ecological conditions interacting with the genetic background of the samples. These components could be associated with the biofunctional properties and they can also affect the organoleptic characteristics of the Piquin chili fruits [34].

### 2.3. Capsaicinoids and SHU

The analysis of capsaicinoids showed significant differences among populations and within each population. The average percentages of capsaicin and dihydrocapsaicin (C:DHC) in the populations were 65.2% and 34.8%, respectively. The capsaicin content was higher than that of dihydrocapsaicin in the three populations analyzed (Table 3). The ratios of these capsaicinoids showed that capsaicin was the major compound with contents that were 1.4 to 2.5 times greater than that of dihydrocapsaicin. This comparison highlighted the fact that the samples from Population II had the lowest C:DHC ratios of all the analyzed samples. 

The significance of differences between mean values was determined by an analysis of variance, which indicated that Population I (15.6 mg/g DW (Dry weight)) had the highest capsaicin content, followed by Populations II (11.5 mg/g DW) and III (mg/g DW). All of the populations had different average contents of dihydrocapsaicin. Thus, the average variation in dihydrocapsaicin was lower (11.2%) than that of capsaicin (15.2%). Using the Tukey’s Honestly Significant Difference (HSD), the significant differences among the means revealed significant variation within the populations. The ecotypes of Population II were similar to accession III-1, and the capsaicin content in III-2 was similar to that of the Population I ecotypes. Additionally, the dihydrocapsaicin content in III-1 and III-2 was similar to that in II-1. All of the populations had different capsaicin contents. The greatest variation of this capsaicinoid within a population was observed within Population III, but the dihydrocapsaicin content was the same between ecotypes. 

The total capsaicinoid content (CAPT) was determined by adding the values of capsaicin and dihydrocapsaicin, which were the most abundant capsaicinoids and contributed the most to the pungency. Differences in the CAPT were associated with the geographic origin of the population. The average CAPT was higher in Population I, which had a CAPT of 23.5 mg/g with a CV of 14.0%, than in Populations II and III, which had average CAPTs of 15.3 and 10.7 mg/g, respectively.

Capsaicinoid contents (ppm) were converted to Scoville pungency heat units (SHU), because that scale is regarded as the best indicator of pungency [45] and it is directly related to CAPT levels. Therefore, the chili populations that had high total capsaicinoid content had high SHU values. Significant differences in pungency among the populations were identified. The Population I showed high levels of variability (30,693.9 to 41,039.3 SHU), with a population average of 38,115.3 SHU. These values were higher than those observed in Populations II and III (25,495.1 and 27,081.4 SHU, respectively). The greatest variation within a population was observed in Population III; this was equivalent at 9781.4 SHU, which was in contrast to the narrow distribution observed in Population II. The pungency values of Population III overlapped with those of Population I and Population II. The SHU means of Populations II and III were proportional and very similar, although there were significant differences between them.

The wild populations of Piquin chili from northern Mexico have higher capsaicinoid contents than those reported for wild populations of Piquin chili from southern Mexico [15]. Differences in the capsaicinoid contents may be associated with genotype, physiological maturity, environmental conditions, or geographic origin [46]. However, Gurung Techawongstien, Suriharn, and Techawongstien [47] suggested that the expression and content of capsaicinoids are closely related to the genetic background of the plant. Nevertheless, the impact of the adaptations of each population to local growing conditions should be considered. 

These adaptations may have resulted in the different capsaicinoid contents of the northern and southern populations. For example, when northern populations are compared, the values of capsaicin and dihydrocapsaicin are similar in populations of Chiltepín chili (regional name for *Capsicum annuum* var. *glabriusculum*) from northeastern Mexico, where the conditions are generally drier than in the southern region [8]. That is, in terms of pungency, the northern populations of Piquin chili are similar, although they are not markedly different from the southern populations. In this sense, Votava, Nabham, and Bosland [48] noted that geographically close wild chili populations are more similar because of factors, such as self-pollinating, a factor that can partially impact the characteristic patterns of each ecotype. Nevertheless, we observed that within each population the pungency varied. Valiente-Banuet, and Gutiérrez-Ochoa [49] observed a positive correlation between increased capsaicinoids and water deficit. 

Our study, however, found different values, even though the ecotypes are from semi-arid and subtropical regions of Tamaulipas. Soil nutrition and fertilization also affect the synthesis and accumulation of capsaicinoids [50], which varies with chili ecotype and variety, especially in wild ecotypes [8]. Moreover, an increase in the concentration of capsaicinoids has been associated with heat stress, which elicits particular responses from each chili type and cultivar, and for this reason, there is broad variability in pungency both among and within ecotypes [51]. The increase in capsaicinoids has been proposed as a survival strategy, highlighting the potential of these metabolites as chemical defenders protecting the seed against attack by pathogens [52]. 

It has been reported that the pungency of Piquin chili varies from 30,000 to 40,000 SHU [48]. However, the values that were found in the populations of this study were higher and more varied. According to Weiss [53], 22% of the accessions analyzed were classified as hot on the pungency scale (3000 to 25,000 SHU) and the rest of the wild chilis were classified as very hot on the pungency scale (25,000 a 70,000 SHU). Although the native populations of Piquin chili were classified in only two categories, there were wide intervals within each category. The levels of pungency in our study varied among the populations and ecotypes of each population. This variation may be associated with genetic characteristics and be indicative of the particular environment of each micro-region where the samples were collected, which influenced the accumulation of metabolites as part of the population’s adaptations to its particular surroundings. 

Although Piquin chili is characterized by high capsaicinoid contents, there were also substantial variations that resulted in the heterogeneity of this organoleptic property. Variations in capsaicinoids and other metabolites depend on adaptations to a particular environment; thus, the geographic variation of species and populations of chili is considered to be an adaptive strategy to cope with biotic and abiotic pressures [5]. 

Plant biochemical plasticity is a survival strategy that includes the activities of many metabolites, and particularly in chili fruits, many of those metabolites of ecological importance are related to sensorial characteristics and their anthropocentric and industrial uses. The results of our study also suggest that the broad variation of the compositional patterns of phytochemical accumulation in natural populations of Piquin chili was related to geographic and ecological differences of the sites where the samples were collected. Finally, knowledge of production patterns, influential factors, and responses could form a foundation for the exploration of ecotypes and microenvironments that would lead to production guided by consumer requirements and avoid excess extraction pressure on wild populations.

## 3. Materials and Methods 

### 3.1. Plant Material and Sample Preparation

From August to September 2016, samples of ripe fruits from wild Piquin chili populations were collected from different geographic locations and environments in the central region of Tamaulipas, Mexico. Three populations (ID = I to III) were sampled with different numbers of ecotypes (4, 3, and 2 accessions, respectively) in each population (Table 4). Samples from each population are indicated by the numbers following the hyphen. All the collected fruits were washed with water and common soap and then rinsed with abundant distilled water. They were then stored in paper bags and dried in a drying convection oven (Jeio Tech, Geumcheon-gu, Seoul, Korea) (30 ± 5 °C for 96 h). After drying, the seeds were removed, and the placenta and pericarp were pulverized in an electric mill (Krups GX4100^®^, Mexico City, Mexico). The samples were stored in polypropylene tubes at −20 °C until extraction.

### 3.2. Methods of Extraction

Water and 70% ethanol were each used as extraction solvents. For each sample, 0.5 g of chili material was mixed with either distilled water or 70% ethanol in a 1:6 ratio (sample: solvent). The mixture was kept at −4 °C for 20 min and vortexed every 5 min. The resulting mixture was centrifuged at 9200 times gravity (× *g*) for 5 min. The supernatant was then recovered, mixed with cold acetone (1:3, *v*/*v*) at −20 °C, and then centrifuged again at 9200× *g* to obtain the pellet by precipitation of polar compounds due to their low solubility in the saturated solvent with acetone [54]. The sample pellet was re-suspended in either water or 70% ethanol for polyphenol and free radical-scavenging capacity analyses.

### 3.3. TPC

The TPC was determined with the Folin-Ciocalteu method [55]. After adding 250 μL of 1 N Folin-Ciocalteu reagent to a 500 µL sample or standard, the mixture was incubated for 5 min, and then 1250 μL of 20% Na_2_CO_3_ was added. The mixture was then left to stand for 2 h, after which the absorbance was read at 750 nm in a spectrophotometer (UV-6000, Metash instruments Co. Ltd., Shanghai, China). A gallic acid (3,4,5-trihydroxybenzoic acid) calibration curve was prepared using a 0.1 mg mL^−1^ solution. The results are expressed in mg of equivalent gallic acid per g of dry weight (mg GAE g^−1^ dry weight).

### 3.4. Antioxidant Capacity

The DPPH^•^ radical scavenging activity was determined following the Brand–Williams procedure [56]. A mixture of 975 µL of 600 µM DPPH^•^ radical in methanol and 25 µL of each extract was prepared. The reaction was left in the dark at 25 ± 2 °C for 30 min after which the absorbance was measured at 515 nm. For quantification, a standard curve of Trolox in concentrations ranging from 0 to 1200 µM was prepared. The results are reported in mM Trolox equivalents (TE) per gram of dry weight. 

Evaluation of the ABTS^•+^ free radical-scavenging activity was conducted following the procedure that was described by Re, Pellegrini, Proteggente, Pannala, Yang, and Rice [57]. A stock solution of a mixture of 7 mM ABTS^•+^ and 2.45 mM potassium persulfate was incubated for 16 h at 25 ± 2 °C in darkness. The solution was later adjusted to 0.7 absorbance at 732 nm to afford the working solution. A 10 µL aliquot of each extract was mixed with l mL of ABTS^•+^ working solution, and the absorbance was recorded at the beginning and end of the reaction (after 6 min). The 4 mM Trolox solution was used to prepare the calibration curve for determining the free radical-scavenging capacity. The scavenging of free radicals was expressed in mM Trolox equivalents (TE per gram of dry weight).

### 3.5. UPLC-ESI-Q/TOF-MSe Analysis

The present compounds were identified using an Acquity ultra-performance liquid chromatography (UPLC) system attached to an auto-sampler and a binary pump with a 10 µL loop. Chromatographic separations were conducted following the methodology that was proposed by Kumari, Elancheran, Kotoky, and Devi [58], with slight modifications using a BEH PHENYL (2.1 mm × 100 mm, 1.7 µm; WATERS, Elstree, Herts UK) analytical column at 40 °C. The compounds in the various extracts were identified by using their full mass spectra and their unique mass fragmentation patterns. Comparison of the observed MS spectra with those found in the literature and databases, such as MassBank (http://www.massbank.jp/), ChemSpider (http://www.chemspider.com), and PubChem (https://pubchem.ncbi.nlm.nih.gov), was the main tool for the identification of the compounds.

### 3.6. Capsaicinoid Analysis

Determination and quantification of capsaicin and dihydrocapsaicin was achieved with the technique described by Collins, Wasmund, and Bosland [59]. The injection volume was 20 µL, the column was a Hypersil ODS C18 column (25 cm × 4.6 mm, 5 µm), the mobile phase consisted of a gradient of acetonitrile: water (45:55), the flow rate was 1.5 mL/min, the runtime was 20 min, and Agilent 1260 Infinity (Agilent Technologies, Santa Clara, CA, USA) HPLC system was used. The capsaicinoids were identified by a comparison of their retention times to those of the capsaicin (natural capsaicin) and dihydrocapsaicin (*Capsicum* sp., ~90%) standards (SIGMA-ALDRICH, St. Louis, MO, USA). The calibration curve was generated with 0.25, 0.5, 1.0, 2.0 mg/mL solutions of each standard, and the capsaicinoid content in parts per million (ppm) was converted to the Scoville scale (SHU) with the formula (Equation (1)) that was proposed by Topuz, and Ozdemir [60]:

SHU = [(Capsaicin (ppm) + dihydrocapsaicin (ppm)) × 16.1] (1)

The ratio between the major capsaicinoids (C:DHC) was obtained by dividing the capsaicin and dihydrocapsaicin contents (mg/g PS) by the total capsaicinoid content.

### 3.7. Statistical Analysis

Analyses of the TPC, free radical-scavenging capacity and capsaicinoid content were performed in triplicate. The one-way ANOVA, comparison of arithmetic means (Tukey *p* ≤ 0.05), and correlation analysis were carried out with SAS V 9.3 (SAS Institute, Cary, NC, USA) [61]. 

## 4. Conclusions

Quantification of phenolic compounds, free radical-scavenging capacities, and capsaicinoid content showed that the phytochemical compositions of wild populations of Piquin chili varied widely among populations and among their ecotypes. Population I presented the highest values for phenolic and capsaicinoid contents, while Population II showed the highest free radical-scavenging capacity, followed by Population III. Population III showed similar phenolic and pungency values to those of Population I, which demonstrated the presence of different combinations of phytochemicals and differential accumulations of free radical-scavenging capacities in each population that could be attributed to the plasticity of each wild ecotype in their particular environments.

Differences in the free radical-scavenging capacity are a result of the differential contributions of each identified compound, whose presence or absence produces a mosaic of phytochemical and functional capacities in each ecotype. However, more detailed studies are needed to understand the individual contributions and physiological roles of each compound.

Based on these results, several new applications and natural resource management strategies for the native populations of Piquin chili from Tamaulipas can be established, because they constitute a wild resource that could be exploited for its phytochemicals. Therefore, the results obtained herein help to elucidate the phytochemical composition of natural populations of Piquin chili.

## Figures and Tables

**Table 1:** Inter- and intra-population phenols variation and free radical-scavenging capacities of wild Piquin chili in aqueous extracts and hydroalcoholic extracts.

Ecotype	Aqueous Extracts			Hydroalcoholic Extracts		
	TPC	DPPH^•^	ABTS^•+^	TPC	DPPH^•^	ABTS^•+^
I-1	188.5 ± 8.6 ^A^	29.8 ± 0.7 ^A^	95 ± 0.8 ^A^	272.3 ± 7.1 ^b^	57.9 ± 0.9 ^b^	125 ± 0.5 ^a^
I-2	137.8 ± 10.2 ^B^	17.4 ± 2.3 ^C^	60.4 ± 1.1 ^B^	309.8 ± 7.9 ^a^	62.6 ± 3.3 ^a^	124.8 ± 0.7 ^a^
I-3	129.8 ± 3.2 ^B^	22.8 ± 0.5 ^B^	40 ± 0.4 ^C^	252.8 ± 4.6 ^b^	53.2 ± 0.2 ^ab^	110 ± 5.7 ^b^
I-4	127.2 ± 1.6 ^B^	22 ± 0.9 ^B^	39.8 ± 6.3 ^C^	263.7 ± 5.1 ^c^	55.6 ± 1.6 ^c^	115 ± 4.2 ^b^
HSD	18.1	3.4	8.4	16.5	5	9.3
Population mean	145.8 ± 26.7	23 ± 4.8	58.8 ± 23.6	274.6 ± 23	57.3 ± 4.0	118.7 ± 7.4
II-1	271.9 ± 15.8 ^B^	1.1 ± 0.4 ^B^	124.7± 0.4 ^B^	515.2 ± 6.0 ^b^	82.5 ± 1.0 ^c^	287.5 ± 4.0 ^a^
II-2	183.4 ± 3.0 ^C^	8.6 ± 0.6 ^A^	117.2 ± 1.9 ^C^	544.6 ± 1.1 ^a^	100 ± 0.3 ^a^	188.3 ± 6.5 ^c^
II-3	353 ± 16.8 ^A^	1.4 ± 0.3 ^B^	189.7 ± 2.4 ^A^	434.4 ± 8.3 ^c^	88.6 ± 1.1 ^b^	263.7 ± 12.4 ^b^
HSD	33.7	1.2	4.5	14.9	2.1	21
Population mean	269.5 ± 74.4	3.7 ± 3.7	143.9 ± 34.6	498 ± 49.7	90.4 ± 7.7	246.5 ± 45.4
III-1	174.3 ± 8.7 ^A^	8.4 ± 0.5 ^B^	117.2 ± 1.9 ^A^	497.9 ± 17.5 ^a^	90.3 ± 1.4 ^a^	283.9 ± 2.5 ^a^
III-2	106.4 ± 1.1 ^B^	12.4 ± 1.0 ^A^	20.2 ± 1.7 ^B^	338.4 ± 17.9 ^b^	41.6 ± 0.7 ^b^	126 ± 0.8 ^b^
HSD	14	1.8	4.1	40.1	2.5	4.2
Population mean	140.3 ± 37.6	10.4 ± 2.3	68.7 ± 53.2	418.1 ± 88.8	66 ± 26.7	204.9 ± 86.5

TPC = mg GAE/g dry weight (DW); DPPH^•^ = mM TE/g DW; ABTS^•+^ = mM TE/g DW. Values in the same column and population with different letters are significantly different (Tukey, *p* ≤ 0.05); ± Standard deviation (SD, *n* = 3). Tukey’s Honestly Significant Difference (HSD).

**Table 2:** Compounds in wild Piquin chili populations identified by LC-MSe.

Peak N°	Rt (min)	[M − H]^−^ (*m*/*z*)	Tentative Assignment	Molecular Formula	MS2 Dominant Fragments Ions	Compound Type	Ecotype (Population-Sample)									Reference
							I-1	I-2	I-3	I-4	II-1	II-2	II-3	III-1	III-2	
1	0.846	191.0874	Quinic acid	C_7_H_11_O_6_	-	Phenolic acid	x				x	x	x	x		[34]
2	1.116	191.0492	Citric acid	C_6_H_8_O_7_	-	Organic acid		x								[34]
3	1.15	180.0985	Tyrosine	C_9_H_10_NO_3_	163.0791	Amino acid	x			x	x	x	x	x	x	[34]
4	1.218	383.1263	Kaempferol-7,4′-Dimethoxy-8-Butyryl ester	C_21_H_20_O_7_	-	Flavonoid	x			x	x	x				[35]
5	1.252	312.0973	1,2,4-trihydroxynonadecane	C_19_H_38_O_3_	313.0905, 225.1853, 279.1969, 293.2083	Fatty alcohol		x					x	x	x	[36]
6	1.319	169.0607	Gallic acid	C_7_H_6_O_5_	125.0751	Phenolic acid			x							*
7	1.421	282.0933	Guanosine	C_10_H_13_N_5_O_5_	150.0444	Nucleoside		x			x	x	x	x	x	[37]
8	1.522	297.1259	Genistein-4,7′-dimethyl ether	C_17_H_14_O_5_	-	Flavonoid	x	x								[35]
9	1.725 2.165	174.9907	Ascorbic acid Isomer	C_6_H_7_O_6_	-	Vitamin	x		x					x		[37]
10	2.03	164.108	dl-Phenylalanine	C_9_H_10_NO_2_	147.044	Amino acid	x	x		x	x		x		x	[34]
11	2.842	301.1075	Quercetin	C_15_H_10_O_7_	173.1554	Flavonoid							x	x		[38]
12	2.909	233.1501	Unknown	-	-	-					x	x				-
13	3.282	203.1112	Tryptophan	C_11_H_11_N_2_O_2_	-	Amino acid	x									[34]
14	3.315	203.1091	7-Ethoxy-4-methylcoumarin	C_12_H_12_O_3_	-	-		x		x	x	x	x	x	x	[35]
15	3.349 3.484	310.1175	Benzopyrano [4,3-β] quinoline-6-ones	C_21_H_13_NO_2_	-	Alkaloid			x							[39]
16	3.417	329.086	Vanillic acid 1-*O*-B-glucopyranosyl ester	C_14_H_18_O_9_	-	Phenolic acid	x	x	x	x	x		x	x	x	[34]
17	3.518	331.1016	1-*O*-galloyl-β-d-glucose	C_13_H_16_O_10_	-	Phenolic acid	x									[40]
18	3.552 3.721	311.0905	2-Caffeoyl-l-tartaric acid	C_13_H_12_O_9_	133.0135, 115.0034	Phenolic acid		x		x						[38]
19	3.958	337.1437	Coumaroyl quinic acid I or II	C_16_H_17_O_8_	191.0571	Phenolic acid	x	x	x	x	x				x	[37]
20	4.161	263.1557	Spinochrome A	C_12_H_8_O_7_	235.9501, 207.9648	-									x	[38]
21	4.702	325.0885	Coumaryl-hexoside	C_15_H_18_O_8_	163.0762	Phenolic acid	x	x	x	x			x		x	[37]
22	4.736	325.0894	Coumaric acid hexose	C_15_H_17_O_8_	163.0762	Phenolic acid					x	x		x		[37]
23	4.838	457.1463	6′′-*O*-Acetyldaidzin	C_23_H_21_O_10_	-	Flavonoid		x								[35]
24	4.872	457.1479	Unknown	-	-	-					x					-
25	5.075	503.1013	Quercetin-3-*O*-(6′′-*O*-acetyl)-β-d-glucopyranoside	C_23_H_21_O_13_	-	Flavonoid	x								x	[35]
26	5.075	653.0688	Quercetin 3,7-diglucuronide	C_27_H_26_O_19_	-	Flavonoid		x								[35]
27	5.108 5.446	563.0996	Apigenin 6-*C*-hexoside-8-*C*-pentoside Isomer	C_27_H_28_O_14_	545.0913, 503.1136	Flavonoid			x							[41]
28	5.244	355.0937	Feruloyl-β-d-glucose	C1_6_H_20_O_9_	175.0408	Phenolic acid	x	x		x	x	x	x			[40]
29	5.278	567.1243	Polygala xanthone III	C_25_H_28_O_15_	-	Xanthone			x					x	x	[42]
30	5.379	352.0956	4-*O*-caffeoylquinic acid	C_16_H_18_O_9_	134.8	Phenolic acid	x	x	x	x	x				x	[43]
31	5.447	431.1585	Apigenin 8-*C*-glucoside	C_21_H_19_O_10_	311.0561	Flavonoid	x	x	x	x	x	x	x	x	x	[44]
32	5.514	374.0412	Unknown	-	-	-	x			x	x	x	x			-

x Present in the ecotype. * Identification confirmed by commercial standard. – Not available information.

**Table 3:** Capsaicinoid content in wild populations of Piquin chili.

Ecotype (Population-Sample)	Capsaicin	Dihidrocapsaicin	Total Capsaicinoid Content	C:DHC	SHU
I-1	16.8 ± 0.2 ^b^	8.4 ± 0.6 ^a^	25.2 ± 0.7 ^a^	2.0:1	41,039.3 ± 1145.7 ^a^
I-2	12.5 ± 0.1 ^d^	6.4 ± 0.05 ^b^	19.0 ± 0.1 ^c^	2.0:1	30,693.9 ± 232.2 ^c^
I-3	18.6 ± 0.2 ^a^	8.6 ± 0.1 ^a^	27.2 ± 0.3 ^a^	2.2:1	44,034.9 ± 465.2 ^a^
I-4	14.6 ± 0.3 ^c^	8.0 ± 0.2 ^a^	22.6 ± 0.5 ^b^	1.8:1	36,693.3 ± 874.5 ^b^
Population mean	15.6	7.9	23.5		38,115.3
HSD	1.0	1.3	2.2		3469.6
CV%	15.3	12.5	14.0		14.1
II-1	8.8 ± 0.1 ^b^	5.3 ± 0.05 ^b^	14.1 ± 0.1 ^b^	1.7:1	22,867.2 ± 227.6 ^b^
II-2	9.7 ± 0.3 ^a^	6.7 ± 0.2 ^a^	16.4 ± 0.5 ^a^	1.4:1	26,460.6 ± 783.1 ^a^
II-3	9.8 ± 0.05 ^a^	6.9 ± 0.03 ^a^	16.7 ± 0.1 ^a^	1.4:1	27,157.4 ± 38.7 ^a^
Population mean	11.5	6.3	15.7		25,495.1
HSD	0.8	0.5	1.3		2045.2
CV%	5.9	12.1	8.3		8.3
III-1	8.9 ± 0.05 ^b^	4.8 ± 0.01 ^b^	13.7 ± 0.1 ^b^	1.9:1	22,190.7 ± 111.3 ^b^
III-2	14 ± 0.4 ^a^	5.6 ± 0.2 ^a^	19.5 ± 0.6 ^a^	2.5:1	31,972.1 ± 917.6 ^a^
Population Mean	9.4	5.2	16.6		27,081.4
HSD	1.1	0.4	1.5		2566.3
CV%	24.4	9.1	19.7		20.1

Average value (mg/g DW) ± Standard deviation (*n* = 3); C:DHC = capsaicin dihydrocapsaicin ratio; SHU = Scoville pungency heat units; CV = coefficient of variation. Different lower-case letters indicate significant differences (*p* ≤ 0.05). Tukey’s Honestly Significant Difference (HSD).

**Table 4:** Origin and environment of wild populations of *C. annuum* var. *glabriusculum* from Tamaulipas, Mexico.

Population	Ecotype (Population-Sample)	Place	Environment			
			Geographic Location (Decimal Grades)		Altitude (m)	Vegetation Type, Clime, Precipitation and Soil
I	I-1	Llera	23.2781 N	−99.0681 W	414	Annual rainfed agriculture; Aw1; Precipitation in the driest month less than 60 mm; Vertisol.
	I-2		23.2761 N	−99.0844 W	403	Secondary vegetation of low deciduous forest; Aw1; Precipitation in the driest month less than 60 mm; Regosol.
	I-3		23.2825 N	−99.0635 W	503	Annual rainfed agriculture; (A) C (wo); Precipitation in the driest month less than 40 mm; Vertisol.
	I-4		23.2824 N	−99.0624 W	504	
II	II-1	Hidalgo	24.1258 N	−99.2569 W	242	Permanent irrigation agriculture; (A) C (wo); Precipitation in the driest month less than 40 mm; Vertisol.
	II-2		24.1255 N	−99.2567 W	249	
	II-3		24.1143 N	−99.2496 W	232	
III	III-1	Soto La Marina	23.7927 N	−98.1129 W	30	Annual irrigation agriculture; BS1 (h′) w; Summer showers; Rendzine.
	III-2		23.8073 N	−98.0352 W	60	Cultivated pasture; (A) C (wo); Precipitation in the driest month of less than 40 mm; Litosol.

## References

[1] Moore B.D., Andrew R.L., Külheim C., Foley W.J. (2014). Explaining intraspecific diversity in plant secondary metabolites in an ecological context. New Phytol..

[2] Chaturvedula V.S.P., Prakash I. (2011). The aroma, taste, color and bioactive constituents of tea. J. Med. Plants Res..

[3] Urbizu-González A.L., Castillo-Ruiz O., Martínez-Ávila G.C.G., Torres-Castillo J.A. (2017). Natural variability of essential oil and antioxidants in the medicinal plant *Turnera diffusa*. Asian Pac. J. Trop. Biomed..

[4] Acunha T.D.S., Crizel R.L., Tavares I.B., Barbieri R.L., Pereira de Pereira C.M., Rombaldi C.V., Chaves F.C. (2017). Bioactive Compound Variability in a Brazilian Pepper Collection. Crop. Sci..

[5] Wahyuni Y., Ballester A.R., Tikunov Y., de Vos R.C.H., Pelgrom K.T.B., Maharijaya A., Sudarmonowati E., Bino R.J., Bovy A.G. (2013). Metabolomics and molecular marker analysis to explore pepper (*Capsicum* sp.) biodiversity. Metabolomics.

[6] Lin C.L., Kang Y.F., Li W.J., Li H.T., Li C.T., Chen C.Y. (2016). Secondary metabolites from the unripe fruits of *Capsicum annuum* var. conoides. Chem. Nat. Compd..

[7] Meckelmann S.W., Riegel D.W., van Zonneveld M., Ríos L., Peña K., Mueller-Seitz E., Petz M. (2015). Capsaicinoids, flavonoids, tocopherols, antioxidant capacity and color attributes in 23 native Peruvian chili peppers (*Capsicum* spp.) grown in three different locations. Eur. Food Res. Technol..

[8] González-Zamora A., Sierra-Campos E., Pérez-Morales R., Vázquez-Vázquez C., Gallegos-Robles M.A., López-Martínez J.D., García-Hernández J.L. (2015). Measurement of capsaicinoids in Chiltepin hot pepper: A comparison study between spectrophotometric method and high-performance liquid chromatography analysis. J. Chem..

[9] Rochín-Wong C.S., Gámez-Meza N., Montoya-Ballesteros L.C., Medina-Juárez L.A. (2013). Efecto de los procesos de secado y encurtido sobre la capacidad antioxidante de los fitoquímicos del chiltepín (*Capsicum annuum* L. var. *glabriusculum*). Rev. Mex. Ing. Quím..

[10] Rodríguez-Maturino A., Valenzuela-Solorio A., Troncoso-Rojas R., González-Mendoza D., Grimaldo-Juárez O., Avilés-Marín M., Cervantes-Diaz L. (2012). Antioxidant activity and bioactive compounds of Chiltepin (*Capsicum annuum* var. *glabriusculum*) and Habanero (*Capsicum chinense*): A comparative study. J. Med. Plants Res..

[11] Baenas N., Beovíc M., Llic N., Moreno D.A., García-Viguera C. (2019). Industrial use of pepper. Capsicum annum L.) derived products: Technological benefits and biological advantages. Food Chem..

[12] Schreiner M. (2005). Vegetable crop management strategies to increase the quantity of phytochemicals. Eur. J. Nutr..

[13] Aguirre-Hernández E., San Miguel-Chávez R., Palma Tenango M., González-Trujano M.E., de la Rosa-Manzano E., Sánchez-Ramos G., Mora-Olivo A., Martínez-Palacios A., Martínez-Avalos J.G. (2017). Capsaicinoids concentration in Capsicum annuum var. glabriusculum collected in Tamaulipas, Mexico. Phyton. Inter. J. Exp. Bot..

[14] Bobinaitė R., Viškelis P., Venskutonis P.R. (2012). Variation of total phenolics, anthocyanins, ellagic acid and radical scavenging capacity in various raspberry (*Rubus* spp.) cultivars. Food Chem..

[15] Vera-Guzmán A.M., Chávez-Servia J.L., Carrillo-Rodríguez J.C., López M.G. (2011). Phytochemical evaluation of wild and cultivated pepper (*Capsicum annuum* L. and *C. pubescens* Ruiz & Pav.) from Oaxaca, Mexico. Chil. J. Agric. Res..

[16] Vega-Gálvez A., Di Scala K., Rodríguez K., Lemus-Mondaca R., Miranda M., López J., Pérez-Won M. (2009). Effect of air-drying temperature on physico-chemical properties, antioxidant capacity, colour and total phenolic content of red pepper (*Capsicum annuum* L. var. *Hungarian*). Food Chem..

[17] Kim J.S., Ahn J., Lee S.J., Moon B., Ha T.Y., Kim S. (2011). Phytochemicals and antioxidant activity of fruits and leaves of paprika (*Capsicum annuum* L.; var. *Special*) cultivated in Korea. J. Food Sci..

[18] Loizzo M.R., Pugliese A., Bonesi M., De Luca D., O’Brien N., Menichini F., Tundis R. (2013). Influence of drying and cooking process on the phytochemical content, antioxidant and hypoglycemic properties of two bell *Capsicum annum* L. cultivars. Food Chem. Toxicol..

[19] Conforti F., Statti G.A., Menichini F. (2007). Chemical and biological variability of hot pepper fruits (*Capsicum annuum* var. *acuminatum* L.) in relation to maturity stage. Food Chem..

[20] Hervert-Hernández D., García O.P., Rosado J.L., Goñi I. (2011). The contribution of fruits and vegetables to dietary intake of polyphenols and antioxidant capacity in a Mexican rural diet: Importance of fruit and vegetable variety. Food Res. Int..

[21] Wangcharoen W., Morasuk W. (2007). Antioxidant capacity and phenolic content of chilies. Kasetsart J. (Nat. Sci.).

[22] Putnik P., Bursác Kovačevíc D., Ježek D., Šustić I., Zorić Z., Dragović-Uzelac V. (2018). High-pressure recovery of anthocyanins from grape skin pomace (Vitis vinifera cv. Teran) at moderate temperature. J. Food Process. Preserv..

[23] Sultana B., Anwar F., Ashraf M. (2009). Effect of extraction solvent/technique on the antioxidant activity of selected medicinal plant extracts. Molecules.

[24] Zhu Z., Jiang T., He J., Barba F.J., Cravotto G., Koubaa M. (2016). Ultrasound-assisted extraction, centrifugation and ultrafiltration: Multistage process for polyphenol recovery from purple sweet potatoes. Molecules.

[25] Ferreira-Zielinski A.A., Isidoro-Haminiuk C.W., Alberti A., Nogueira A., Mottin D.I., Granato D.A. (2014). A comparative study of the phenolic compounds and the in vitro antioxidant activity of different Brazilian teas using multivariate statistical techniques. Food Res. Int..

[26] Durak A., Kowalska I., Gawlik-Dziki U. (2017). UPLC-MS method for determination of phenolic compounds in chili as a coffee supplement and their impact of phytochemicals interactions on antioxidant activity in vitro. Acta Chromatogr..

[27] Teleszko M., Wojdylo A. (2015). Comparison of phenolic compounds and antioxidant potential between selected edible fruits and their leaves. J. Funct. Foods.

[28] Shi Q., Chen J., Zhou Q., Lei H., Luan L., Liu X., Wu Y. (2015). Indirect identification of antioxidants in Polygalae Radix through their reaction with 2,2-diphenyl-1-picrylhydrazyl and subsequent HPLC-ESI-Q-TOF-MS/MS. Talanta.

[29] Hou Y., Vasileva E.A., Carne A., McConnell M., Bekhit A.E.D.A., Mishchenko N.P. (2018). Naphthoquinones of the spinochrome class: Occurrence, isolation, biosynthesis and biomedical applications. RSC Adv..

[30] Nakatani N., Kayano S.I., Kikuzaki H., Sumino K., Katagiri K., Mitani T. (2000). Identification, quantitative determination, and antioxidative activities of chlorogenic acid isomers in prune (*Prunus domestica* L.). J. Agric. Food Chem..

[31] Gülçin I. (2007). Comparison of in vitro antioxidant and antiradical activities of l-tyrosine and l-Dopa. Amino Acids.

[32] Ozgen M., Reese R.N., Tulio A.Z., Scheerens J.C., Miller A.R. (2006). Modified 2, 2-azino-bis-3-ethylbenzothiazoline-6-sulfonic acid (ABTS) method to measure antioxidant capacity of selected small fruits and comparison to ferric reducing antioxidant power (FRAP) and 2,2′-diphenyl-1-picrylhydrazyl (DPPH) methods. J. Agric. Food Chem..

[33] Omar M.H., Mullen W., Crozier A. (2011). Identification of proanthocyanidin dimers and trimers, flavone *C*-glycosides, and antioxidants in Ficus deltoidea, a Malaysian herbal tea. J. Agric. Food Chem..

[34] Morales-Soto A., Gómez-Caravaca A.M., García-Salas P., Segura-Carretero A., Fernández-Gutiérrez A. (2013). High-performance liquid chromatography coupled to diode array and electrospray time-of-flight mass spectrometry detectors for a comprehensive characterization of phenolic and other polar compounds in three pepper (*Capsicum annuum* L.) samples. Food Res. Int..

[35] National Center for Biotechnology Information PubChem Compound Database. https://pubchem.ncbi.nlm.nih.gov/.

[36] Hurtado-Fernández E., Carrasco-Pancorbo A., Fernández-Gutiérrez A. (2011). Profiling LC-DAD-ESI-TOF MS Method for the Determination of Phenolic Metabolites from Avocado (*Persea americana*). J. Agric. Food Chem..

[37] Gómez-Romero M., Segura-Carretero A., Fernández-Gutiérrez A. (2010). Metabolite profiling and quantification of phenolic compounds in methanol extracts of tomato fruit. Phytochemistry.

[38] Abu-Reidah I.M., Ali-Shtayeh M.S., Jamous R.M., Arráez-Román D., Segura-Carretero A. (2015). HPLC–DAD–ESI-MS/MS screening of bioactive components from *Rhus coriaria* L. (Sumac) fruits. Food Chem..

[39] Narodiya V.P., Vadodaria M.S., Vagadiya G.V., Gadara S.A., Ladwa K.D. (2014). Synthesis, characterization and Biological Activiti Studies of substituted 6H-12Hbenzopyrano [4,3-b]quinolin-6-one. IJETR.

[40] Gómez-Caravaca A.M., Segura-Carretero A., Fernández-Gutiérrez A., Caboni M.F. (2011). Simultaneous Determination of Phenolic Compounds and Saponins in Quinoa (*Chenopodium quinoa* Willd) by a Liquid Chromatography Diode Array Detection Electrospray Ionization Time-of-Flight Mass Spectrometry Methodology. J. Agric. Food Chem..

[41] Marín A., Ferreres F., Tomás-Barberán F.A., Gil M.I. (2004). Characterization and Quantitation of Antioxidant Constituents of Sweet Pepper (*Capsicum annuum* L.). J. Agric. Food Chem..

[42] Lv C., Li Q., Zhang X., He B., Xu H., Yin Y., Liu R., Liu J., Chen X., Bi K. (2014). Simultaneous quantitation of polygalantoxanthone III and four ginsenoides by ultra-fast liquid chromatography with tandem mass spectrometry in rat and beagle dog plasm after oral administration of kai-Xin-San: Aplication to a comparative pharmacokinetic study. J. Sep. Sci..

[43] Lee S.H., Jaiswal R., Kuhnert N. (2016). Analysis on different grades of highly-rated Jamaica Blue, Mountain coffees compared to easily available originated coffee beans. SCIREA J. Food.

[44] López-Gutiérrez N., Romero-González R., Martínez-Vidal J.L., Garrido-Frenich A. (2016). Determination of polyphenols in grape-based nutraceutical products using high resolution mass spectrometry. LWT-Food Sci. Technol..

[45] Bhagawati M., Saikia A. (2015). Cultivar variation for capsaicinoid content in some processed products of chilli. J. Hortic. Sci..

[46] Barbero G.F., Liazid A., Azaroual L., Palma M., Barroso C.G. (2016). Capsaicinoid Contents in Peppers and Pepper-Related Spicy Foods. Int. J. Food Prop..

[47] Gurung T., Techawongstien S., Suriharn B., Techawongstien S. (2012). Stability analysis of yield and capsaicinoids content in chili (*Capsicum* spp.) grown across six environments. Euphytica.

[48] Votava E.J., Nabham G.P., Bosland P.W. (2002). Genetic diversity and similarity revealed via molecular analysis among and within an in-situ population and ex situ accessions of chiltepin (*Capsicum annuum* var. *glabriusculum*). Conserv. Genet..

[49] Valiente-Banuet J.I., Gutiérrez-Ochoa A. (2016). Effect of Irrigation Frequency and Shade Levels on Vegetative Growth, Yield, and Fruit Quality of Piquin Pepper (*Capsicum annuum* L. var. *glabriusculum*). HortScience.

[50] Hallmann E., Rembiałkowska E. (2012). Characterization of antioxidant compounds in sweet bell pepper (*Capsicum annuum* L.) under organic and conventional growing systems. J. Sci. Food Agric..

[51] González-Zamora A., Sierra-Campos E., Luna-Ortega J.G., Pérez-Morales R., Ortiz J.C.R., García-Hernández J.L. (2013). Characterization of different capsicum varieties by evaluation of their capsaicinoids content by high performance liquid chromatography, determination of pungency and effect of high temperature. Molecules.

[52] Fricke E.C., Haak D.C., Levey D.J., Tewksbury J.J. (2016). Gut passage and secondary metabolites alter the source of post-dispersal predation for bird-dispersed chili seeds. Oecologia.

[53] Weiss E.A. (2002). Capsicum and chilli. Spice Crops.

[54] Torres-Castillo J.A., Sinagawa-García S.R., Martínez-Ávila G.C.G., López-Flores A.B., Sánchez-González E.I., Aguirre-Arzola V.E., Torres-Acosta R.I., Olivares-Sáenz E., Osorio-Hernández E., Gutiérrez-Díez A. (2013). Moringa oleifera: Phytochemical detection, antioxidants, enzymes and antifungal properties. Phyton. Int. J. Exp. Bot..

[55] Singleton V.L., Orthofer R., Lamuela-Raventós R.M. (1999). Analysis of total phenols and other oxidation substrates and antioxidants by means of Folin-Ciocalteu reagent. Methods Enzimol..

[56] Brand-Williams W., Cuvelier M.E., Berset C.L.W.T. (1995). Use of a free radical method to evaluate antioxidant activity. Food Sci. Technol..

[57] Re R., Pellegrini N., Proteggente A., Pannala A., Yang M., Rice E.C. (1999). Antioxidant activity applying an improved ABTS radical cation decolorization assay. Free Radic. Biol. Med..

[58] Kumari S., Elancheran R., Kotoky J., Devi R. (2016). Rapid screening and identification of phenolic antioxidants in *Hydrocotyle sibthorpioides* Lam. by UPLC–ESI-MS/MS. Food Chem..

[59] Collins M.D., Wasmund L.M., Bosland P.W. (1995). Improved method for quantifying capsaicinoids in *Capsicum* using high-performance liquid chromatography. Hortscience.

[60] Topuz A., Ozdemir F. (2007). Assessment of carotenoids, capsaicinoids and ascorbic acid composition of some selected pepper cultivars (*Capsicum annuum* L.) grown in Turkey. J. Food Compos. Anal..

[61] SAS Institute (2011). SAS User’s Guide: Statistics.

